# ^68^Ga-FAPI PET/CT for diagnostic accuracy and therapeutic response assessment in bleomycin-induced pulmonary fibrosis: an integrated preclinical study

**DOI:** 10.3389/fmed.2025.1613010

**Published:** 2025-06-26

**Authors:** Rui Sun, Yilin Huang, Hao Deng, Qixin Wang, Canran Xiao, Chunmei Guo, Tianyu Wang, Lisheng Liu, Jun Hua, Xiaoliang Chen

**Affiliations:** Department of Nuclear Medicine, Chongqing University Cancer Hospital, Chongqing, China

**Keywords:** ^68^Ga-FAPI-04, bleomycin-induced pulmonary fibrosis, PET/CT, diagnosis, treatment monitoring

## Abstract

**Objective:**

This study aimed to assess the diagnostic and therapeutic monitoring potential of ^68^Ga-fibroblast-activating protein inhibitor (FAPI) positron emission tomography/computed tomography (PET/CT) in bleomycin-induced pulmonary fibrosis (BIPF).

**Methods:**

A preclinical model was established through intratracheal bleomycin administration (2 mg/kg) in C57BL/6 mice, with nintedanib treatment (50 mg/kg/day) initiated at day 28 post-modeling for longitudinal evaluation. Disease progression and therapeutic response were analyzed weekly over 5 weeks using ^68^Ga-FAPI-04 PET/CT, complemented by histopathological validation through fibroblast activation protein (FAP) immunohistochemistry.

**Results:**

In untreated fibrotic mice, ^68^Ga-FAPI-04 uptake demonstrated a progressive increase, peaking at 4w (SUVmean: Left lung: 0.68 ± 0.14; Right lung: 0.65 ± 0.18). Conversely, nintedanib-treated mice exhibited an unexpected elevation in tracer uptake during late-phase imaging, but SUVR showed a decrease than untreated.

**Conclusion:**

These findings underscore ^68^Ga-FAPI-04 PET/CT as a sensitive tool for non-invasive assessment of BIPF early diagnosis and progression. The observed discordance in tracer uptake patterns between treatment groups highlights the need for further investigation into the temporal dynamics of antifibrotic therapy response.

## 1 Introduction

Bleomycin (BLM), an aminoglycoside peptide antibiotic, is a broad-spectrum chemotherapeutic agent that has been clinically utilized for over five decades in the treatment of various neoplastic and non-neoplastic conditions (1). Its unique mechanism of action confers the advantage of lacking immunosuppressive and myelosuppressive side effects, making it particularly promising for the management of hematologic malignancies, especially Hodgkin lymphoma (HL) and germ cell tumors (2). However, due to the lack of BLM hydrolase in the lungs, BLM accumulates in pulmonary tissue during chemotherapy, leading to dose-limiting pulmonary toxicity clinically manifested as pulmonary fibrosis (3–5). The incidence of pulmonary toxicity occurring in 8–10% of patients treated with bleomycin (6, 7). The critical intervention upon the onset of pulmonary toxicity is immediate discontinuation of treatment; however, this adverse event poses significant challenges in clinical management due to the need to balance therapeutic efficacy with irreversible organ damage. Despite advancements in anti-fibrotic therapies such as pirfenidone and nintedanib (8, 9), patient prognosis remains poor, underscoring the urgent need for improved diagnostic and monitoring tools (10).

The primary diagnostic modalities for pulmonary fibrosis include high-resolution computed tomography (HRCT) (11), pulmonary function tests (PFTs), diffusion capacity for carbon monoxide (DLCO) (12), alveolar-arterial oxygen gradient (A-a gradient), MRI, ^18^F-FDG PET/CT, and the gold standard of lung biopsy (13). Nevertheless, these methods show limitations in predicting and monitoring fibrotic activity. While HRCT serves as the cornerstone for diagnosis, it only detects established morphological changes and cannot confirm ongoing tissue remodeling (14). Functional assessments such as PFTs, DLCO, and A-a gradient require long-term follow-up. Mahmutovic Persson et al. have conducted extensive research on MRI imaging of Bleomycin-Induced Pulmonary Fibrosis (BIPF), which is a very promising imaging method (15–19). However, it is currently only applied in animal pulmonary fibrosis models. To date, molecular imaging approaches for evaluating fibrotic activity include ^18^F-FDG PET/CT, ^18^F-FMISO PET/CT and collagen I tracer (15, 18, 20, 21). However, ^18^F-FDG primarily reflects inflammatory activity (22), and ^18^F-FMISO assesses cellular hypoxia (23), collagen I tracer cannot track early-onset fibrosis (15). None of these methods can directly track the presence of activated fibroblasts in tissue.

As there is currently no tool to predict disease progression in BIPF patients, the discovery of noninvasive biomarkers to promote early diagnosis and monitor fibrosis evolution is key to improving patients’ outcomes and therapy efficiency (24). Molecular imaging modalities such as PET/CT have gained traction. The novel tracer ^68^Ga-FAPI-04 PET specifically targets FAP, a biomarker overexpressed in active fibrotic lesions (25). In recent years, FAP, a biomarker overexpressed by cancer-associated fibroblasts, has emerged as one of the most promising biomarkers in oncology (26–28). Bergmann et al. (27) presented the first in-human evidence that fibroblast activation correlates with fibrotic activity and disease progression in the lungs of patients and that ^68^Ga-FAPI-04 PET-CT might improve risk assessment of systemic sclerosis-associated in interstitial lung disease. Similarly, FAP overexpression has been detected in various fibroblast-mediated inflammatory conditions such as liver cirrhosis and idiopathic pulmonary fibrosis (22, 29). ^68^Ga-FAPI-04 PET/CT is a clinically applicable imaging modality that enables direct visualization and quantification of fibroblast-mediated fibrotic activity. This technique holds promise as a non-invasive approach for early prediction and post-therapeutic monitoring of bleomycin-induced pulmonary toxicity.

This study investigates the diagnostic and therapeutic monitoring potential of ^68^Ga-FAPI-04 PET in a BIPF model. We aim to elucidate its unique advantages in capturing dynamic fibroblast activity, ultimately addressing critical gaps in noninvasive BIPF assessment.

## 2 Results

### 2.1 ^68^Ga-FAPI-04 PET detects BIPF

**Figure 1:**
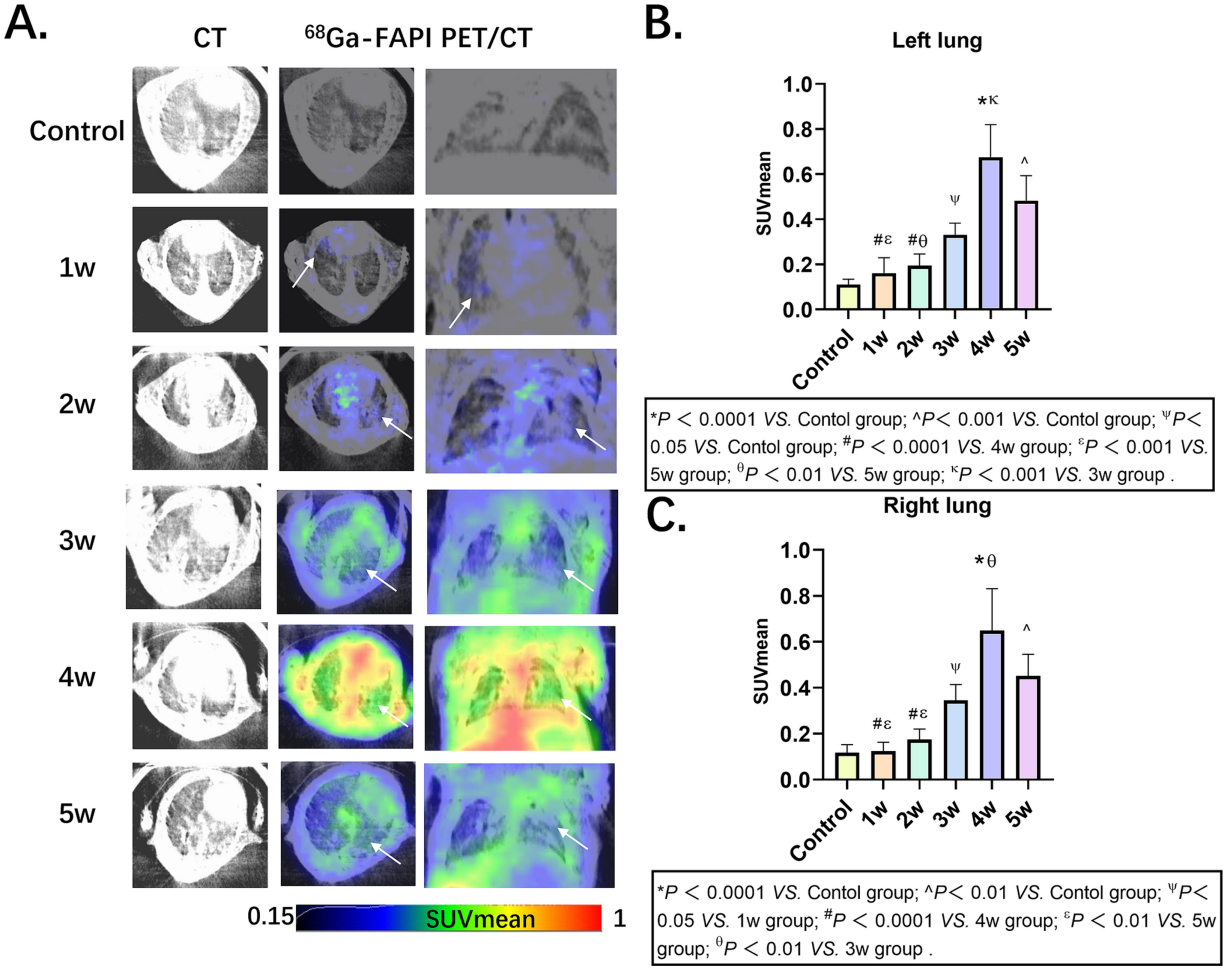
Pulmonary fibrosis murine models in groups Control, 1w, 2w, 3w,4w and 5w of ^68^Ga-FAPI-04, respectively. **(A)** CT and PET/CT images of mice in groups, the arrows indicate regions of elevated ^68^Ga-FAPI-04 uptake. **(B,C)** Comparative quantitative analysis of ^68^Ga-FAPI-04 PET Uptake in control and time-stratified cohorts (1w to 5w post-modeling). Statistical significance was confirmed by one-way ANOVA with Tukey’s *post hoc* test (*p* < 0.05).

PET imaging using ^68^Ga-FAPI-04 enabled the detection of pulmonary fibrosis. Initially, lung uptake regions in the BLM group increased then decreased slowly (Figure 1A), the lung fibrosis was visualized in models. The SUVmean of left lung and right lung in Control, 1w, 2w, 3w,4w and 5w groups as follows: Left lung: 0.11 ± 0.02, 0.16 ± 0.07, 0.19 ± 0.05, 0.33 ± 0.05, 0.68 ± 0.14 and 0.48 ± 0.11, Right lung: 0.12 ± 0.03, 0.12 ± 0.04, 0.18 ± 0.05, 0.35 ± 0.07, 0.65 ± 0.18 and 0.45 ± 0.09 (Figures 1B,C; Supplementary Tables S1, S2).

### 2.2 *Ex vivo* biodistribution of ^68^Ga-FAPI-04 in BIPF murine models

**Figure 2:**
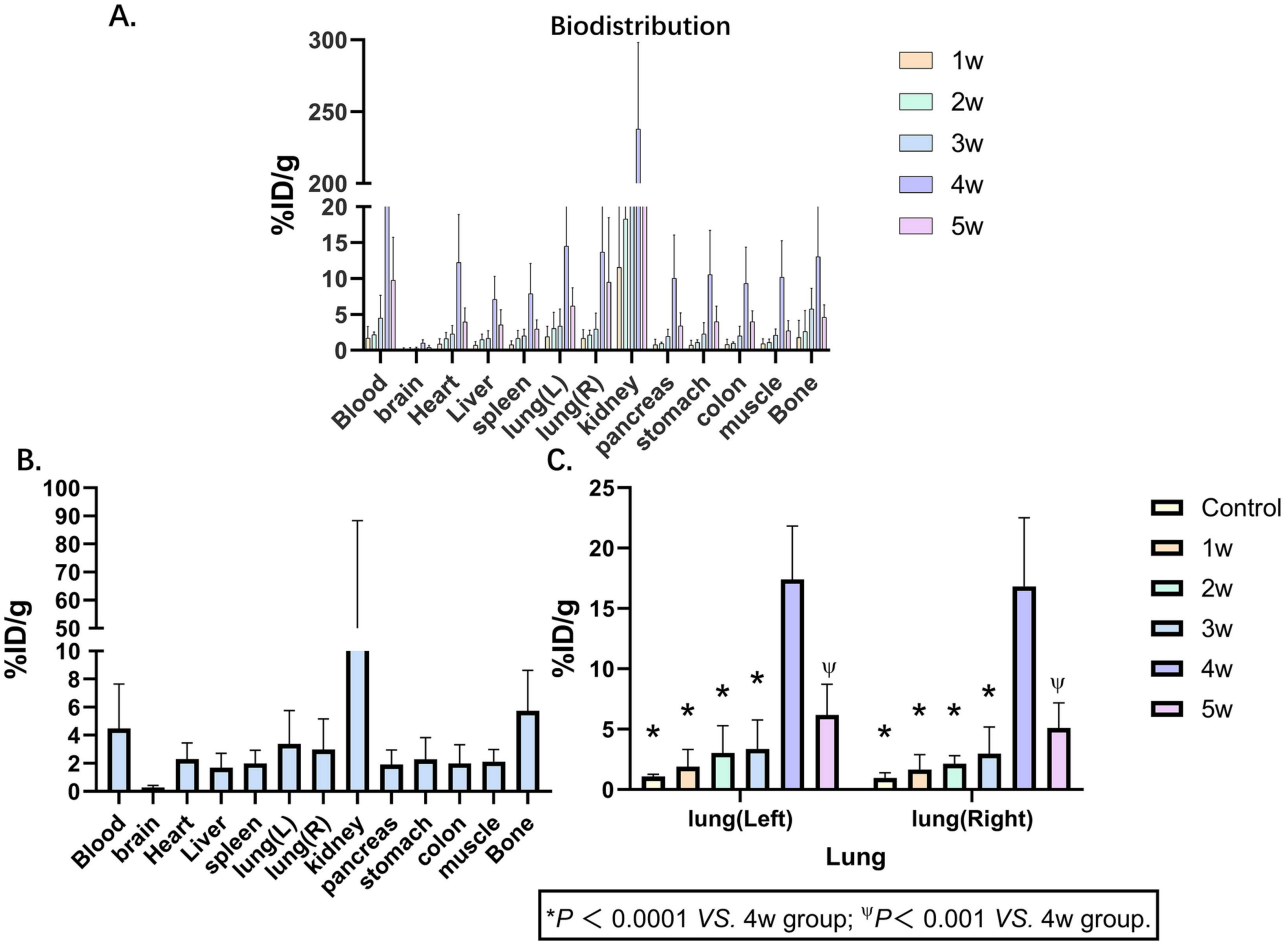
Biodistribution of ^68^Ga-FAPI-04. **(A)** Biodistributions of ^68^Ga-FAPI-04 in pulmonary fibrosis murine models at 1, 2, 3, 4 and 5 weeks show broad distribution in tissues, primarily in the kidney. **(B)** Biodistributions of ^68^Ga-FAPI-04 in pulmonary fibrosis murine models at 3 weeks. **(C)** ^68^Ga-FAPI-04 uptake kinetics in bilateral pulmonary lobes. Statistical significance was confirmed by one-way ANOVA with Tukey’s post hoc test (*p* < 0.05 vs. week 4).

In the BIPF murine models, longitudinal *ex vivo* biodistribution studies were systematically evaluated at 1, 2, 3, 4, and 5 weeks post-modeling. *Ex vivo* organs (blood, brain, heart, liver, spleen, left lung, right lung, kidney, pancreas, stomach, colon, muscle, bone) were performed at 1 h pi. The administered activity (%ID/g) is shown in Figure 2A (Supplementary Table S3). The uptake of various organs shows a trend of rising first and then falling and reaches its peak in the 4th week. This trend is consistent with the results of PET imaging. The *ex vivo* biodistribution was shown in the 3rd week in Figure 2B. The kidney demonstrated the highest radiotracer accumulation (43.97 ± 23.20 %ID/g), whereas the brain exhibited the lowest detectable uptake levels (0.28 ± 0.08 %ID/g) (Figure 2C; Supplementary Tables S4, S5). Delineates the temporal evolution of ^68^Ga-FAPI-04 uptake kinetics in bilateral pulmonary lobes. Longitudinal monitoring revealed dynamic changes in %ID/g (mean ± SD) across control and fibrotic groups from weeks 1 to 5 post-BLM induction. Control group: Left lung 1.07 ± 0.19 %ID/g; Right lung 0.96 ± 0.42 %ID/g; Week 1–5 cohorts: Left lung: 1.89 ± 1.44, 3.04 ± 2.26, 3.37 ± 2.38, 14.49 ± 6.85, 6.18 ± 2.54% ID/g, Right lung: 1.66 ± 1.22, 2.14 ± 0.66, 2.98 ± 2.19, 13.66 ± 7.86, 5.09 ± 2.10 %ID/g.

### 2.3 Pathological findings of BIPF

**Figure 3:**
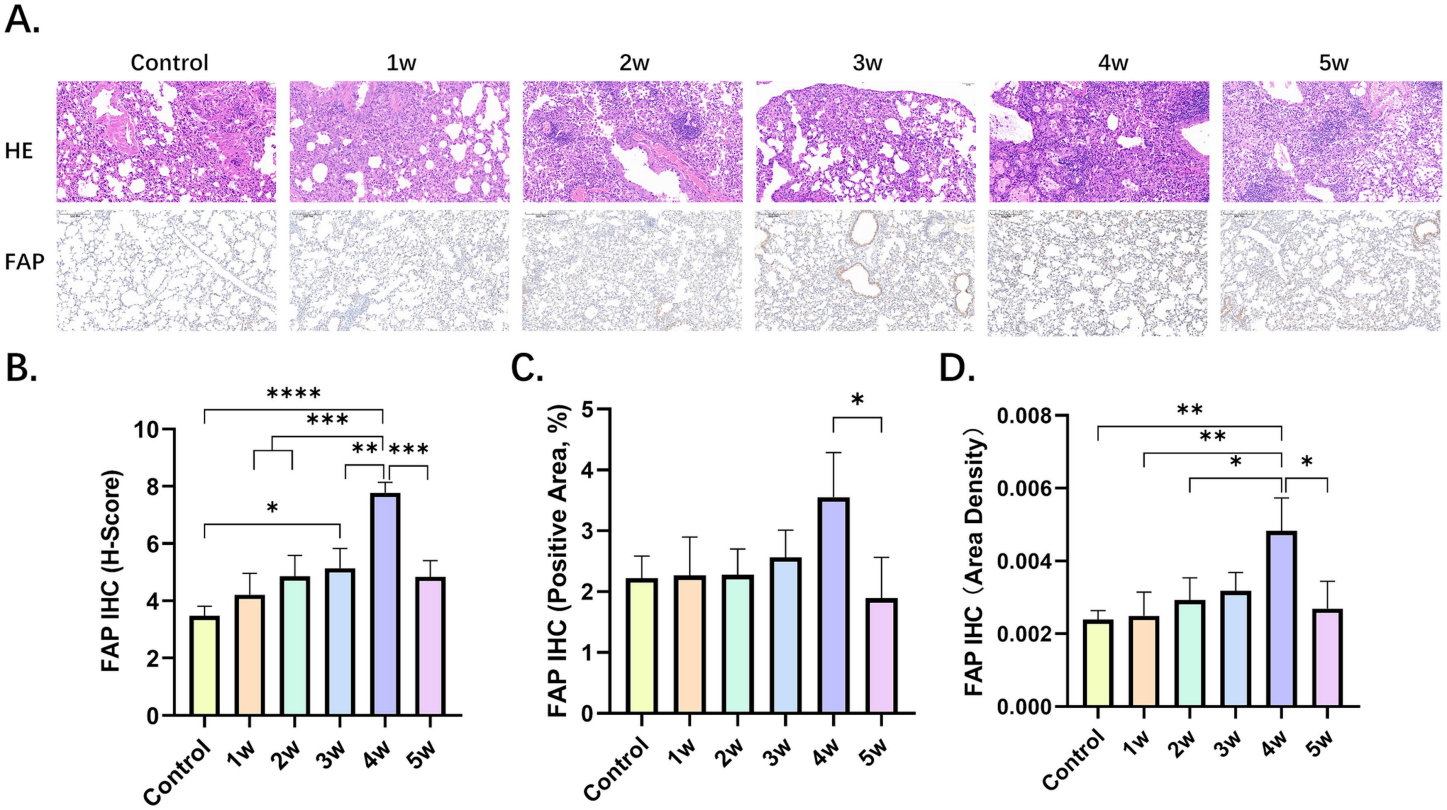
Pathological findings of BIPF. **(A)** Lung hematoxylin–eosin (H&E) staining, IHC of FAP in the control group and BLM group at week 1, 2, 3, 4 and 5. **(B,C)** Quantitative analysis of FAP IHC by H-score **(B)**, positive area ratio **(C)**, and area density **(D)**. Statistical significance was confirmed by one-way ANOVA (*p < 0.05, **p < 0.01, ***p < 0.001, ****p < 0.0001).

In the control group and the BLM group, there were differences in the degree of inflammatory cell infiltration, alveolar epithelial cell hyperplasia, and alveolar collapse (Figure 3A). Fibroblasts stained positive for FAP. We performed a quantitative analysis of FAP immunohistochemistry (IHC) using the Histochemistry score (H-score), positive area ratio, and area density (Figures 3B–D; Supplementary Tables S6–S8). Among the five experimental groups, the 4w exhibited the highest values, with the H-score, positive area ratio, and mean optical density being 7.78 ± 0.37, 3.55 ± 0.73 and 0.48% ± 0.10%, respectively.

### 2.4 Combination therapy with nintedanib reflect by PET and pathological changes

**Figure 4:**
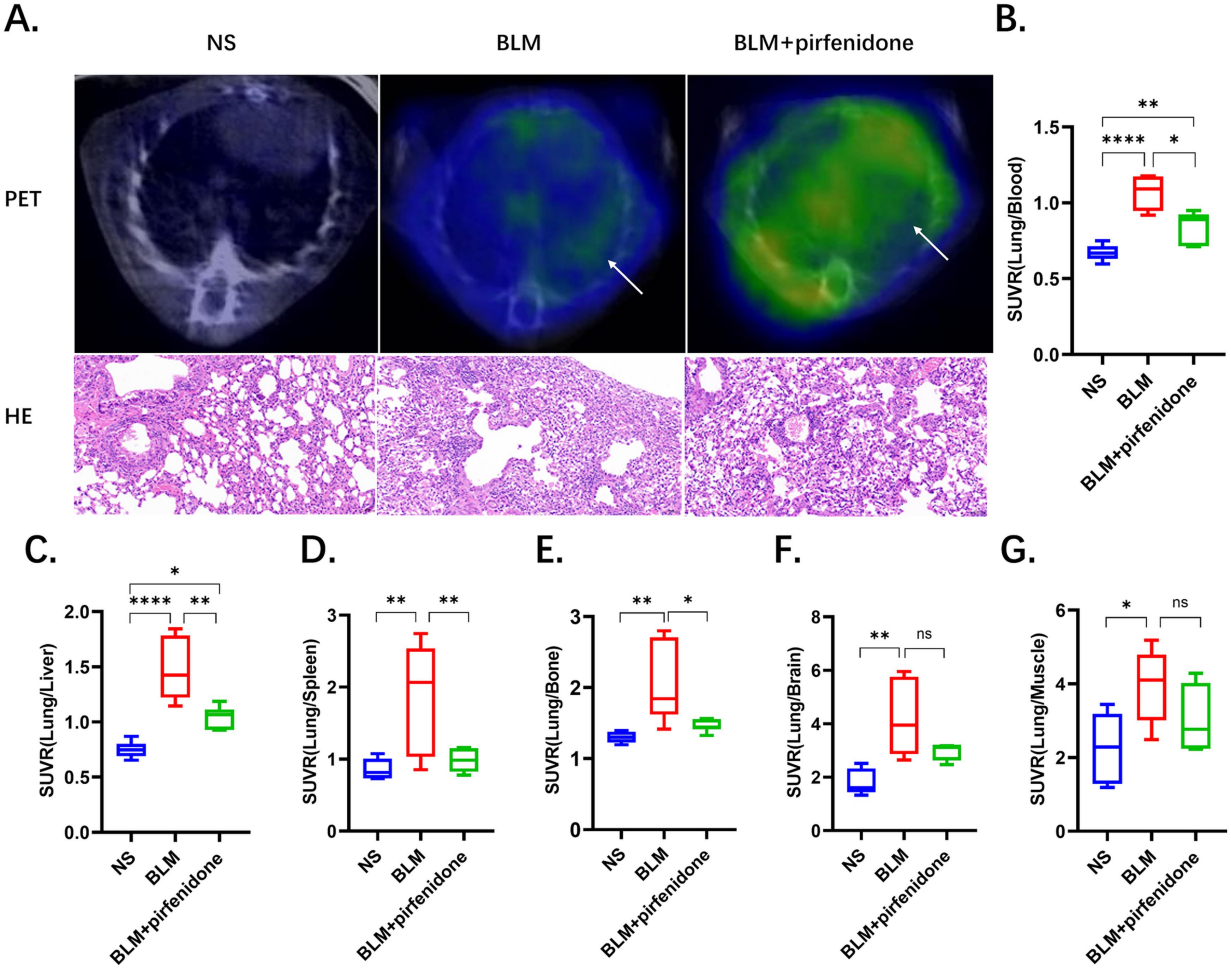
The utilization of ^68^Ga-FAPI PET imaging provides a highly sensitive and noninvasive method to quantitatively assess the efficacy of pirfenidone-based anti-fibrotic therapies in pulmonary fibrosis. **(A)** Comparative ^68^Ga-FAPI-04 PET and HE staining images before and after pirfenidone treatment, the arrows indicate regions of elevated ^68^Ga-FAPI-04 uptake. **(B–G)** SUV ratios were performed for the control, BLM, and BLM + pirfenidone groups. SUV ratios of Lung/Blood **(B)**, Lung/Liver **(C)**, Lung/Bone **(D)**, Lung/Brain **(E)**, Lung/Spleen **(F)**, and Lung/Muscle **(G)** were quantitatively analyzed.

Comparative ^68^Ga-FAPI-04 PET and HE staining images before and after nintedanib treatment are presented in Figure 4A, demonstrating the therapeutic effects on fibrosis in the control, BLM and BLM + nintedanib groups, respectively. Compared with the control group, the lung FAPI uptake was significantly increased before treatment and increased after nintedanib therapy (Supplementary Table S9). The SUVmean was 0.11 ± 0.02, 0.45 ± 0.10 and 0.60 ± 0.11, respectively, among NS, BLM and BLM + nintedanib groups. Furthermore, SUV ratios of Lung/Blood, Lung/Liver, Lung/Bone, Lung/Brain, Lung/Spleen, and Lung/Muscle were quantitatively analyzed (Figures 4B–G; Supplementary Tables S10–S15). In contrast to the SUVmean, all SUV ratios (SUVR) exhibited a decreasing trend following treatment. Moreover, statistically significant differences were observed in the pre-treatment and post-treatment SUV ratios of lung/blood, lung/liver, lung/bone, and lung/spleen. Statistical significance was confirmed by one-way ANOVA (*p < 0.05, **p < 0.01, ***p < 0.001, ****p < 0.0001). Combination therapy with nintedanib significantly inhibits the BLM-induced lung fibrosis reflected by PET and pathological changes.

### 2.5 *In vivo* FAPI-04 target engagement quantification for FAP monitoring via PET-based SUVR analysis

**Figure 5:**
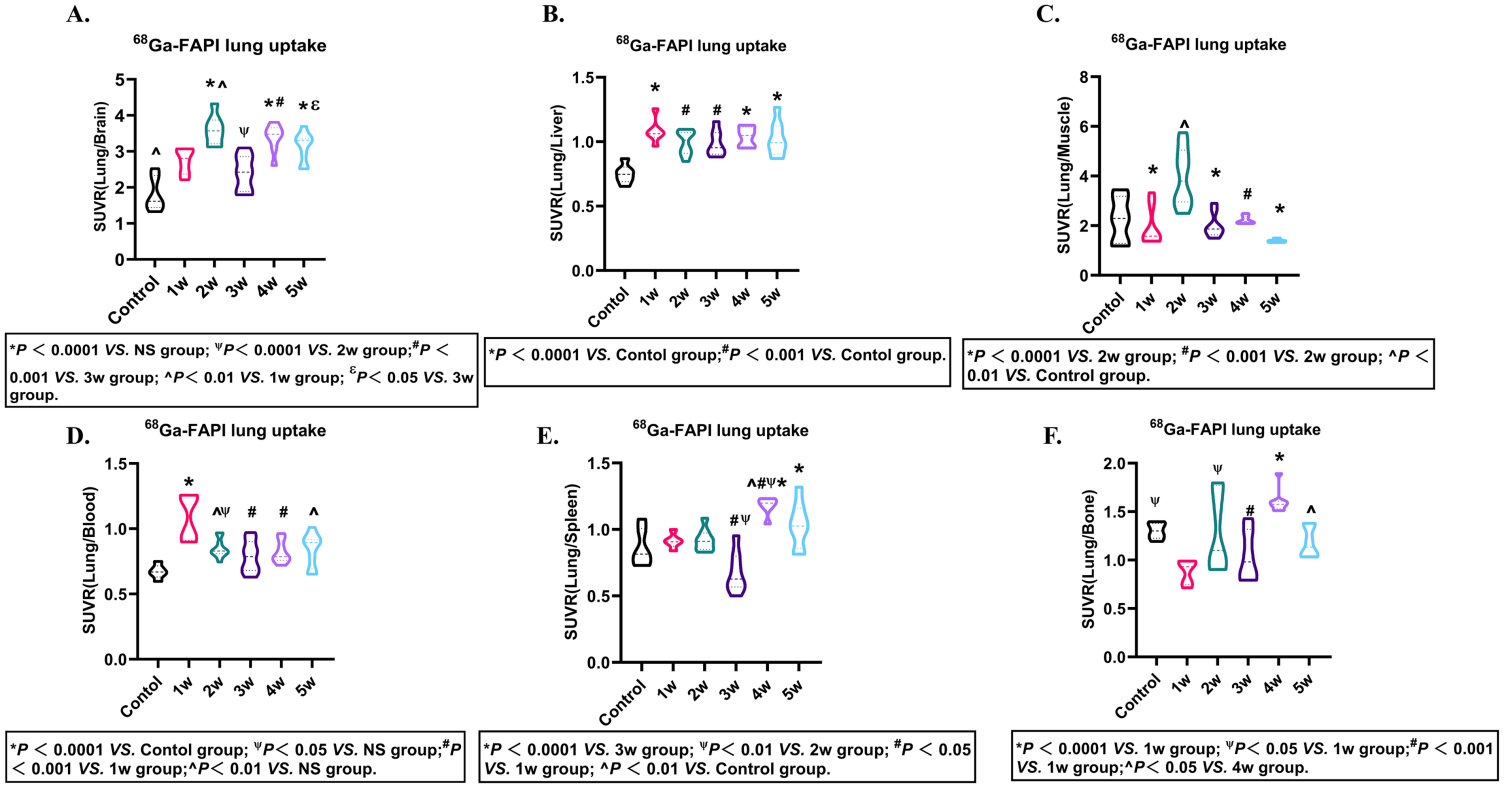
FAPI-04 biodistribution SUVR. Quantitative analysis of SUVR (Lung/Blood) **(A)**, SUVR (Lung/Liver) **(B)**, SUVR (Lung/Bone) **(C)**, SUVR (Lung/Brain) **(D)**, SUVR (Lung/Spleen) **(E)**, SUVR (Lung/Muscle) **(F)** in pulmonary fibrosis murine models. Statistical significance was confirmed by one-way ANOVA with Tukey’s post hoc test (*p* < 0.05).

To further assess the FAPI-04 for the specificity FAP, PET imaging (SUVR) from the pulmonary fibrosis murine models was performed. The outcomes revealed that FAPI-04 exhibited significant binding specificity for pulmonary fibrosis (Figures 5A–F; Supplementary Tables S16–S21). These results demonstrated that SUVR can accurately monitor the progression of pulmonary fibrosis via SUVR, providing a non-invasive and quantitative method for evaluating disease severity and therapeutic response.

## 3 Discussion

For the diagnosis and follow-up of various diseases, *in vivo* molecular imaging has lately emerged as a key tool in preclinical research, clinical trials and clinical practice (20, 22, 24, 30). The present study aims to assess the diagnostic and therapeutic monitoring potential of ^68^Ga- FAPI-04 PET/CT in BIPF, and through ^68^Ga-FAPI-04 PET/CT, *ex vivo* of biodistribution, HE and IHC methods, we have obtained core findings as follows: (a) Longitudinal evaluation of ^68^Ga- FAPI-04 uptake in murine pulmonary fibrosis models demonstrated a characteristic temporal uptake pattern, with maximal tracer accumulation observed at week 4 (Left lung: SUVmax 0.68 ± 0.14, Right lung: SUVmax 0.65 ± 0.18, *p* < 0.05) followed by a statistically significant reduction in uptake values by week 5. (b) The *ex vivo* biodistribution of the lung consisted of *in vivo*. (c) ^68^Ga- FAPI-04 PET/CT imaging was in good agreement with histological findings. (d) ^68^Ga- FAPI-04 PET/CT enables noninvasive monitoring of therapeutic efficacy in pirfenidone-treated pulmonary fibrosis. (e) The multi-compartment SUVR ratios (Lung/Blood, Lung/Liver, Lung/Bone, Lung/Brain, Lung/Spleen, Lung/Muscle) demonstrate significant potential as quantitative biomarkers for noninvasive monitoring of BIPF progression. e. pulmonary fibrosis in patients with different tumors also has FAPI-04 uptake.

Current studies have established the diagnostic and prognostic roles of ^18^F-FDG and ^68^Ga-FAPI PET in pulmonary fibrosis (22, 31–34). In our investigation, longitudinal ^68^Ga-FAPI-04 PET monitoring revealed a biphasic trajectory of radiotracer uptake in bilateral lungs-initial escalation followed by gradual decline. Compared with the work by Ji et al. (22), which compared FAPI PET with CT and ^18^F-FDG while analyzing their correlations, our study uniquely incorporated tumor patients exhibiting pulmonary ^68^Ga-FAPI-04 uptake. Notably, their cohort focused on idiopathic pulmonary fibrosis (IPF), whereas ours centered on bleomycin-induced pulmonary toxicity in cancer patients. Their data indicated peak pulmonary uptake at 21 days post-induction, whereas our murine model demonstrated maximal uptake at 4 weeks. We speculate this discrepancy may stem from delayed fibrogenesis due to transportation-related stress in shipped mice, though this remains hypothetical. Nevertheless, the observed peak window (21–28 days) aligns with most published reports (21, 35). Our research results are consistent with those of Mahmutovic Persson et al. (19). Mechanistically, our research targeted FAP-driven fibrogenesis, contrasting with Dias et al.’s (24) collagen-centric approach. They used ^68^Ga-NODAGA- collagelin as a probe for PET imaging that mainly targets collagen I and III. Collectively, these findings validate the successful establishment of the bleomycin-induced pulmonary fibrosis model in rats, demonstrating robust fibrotic progression during the advanced disease stages. These results further indicate that ^68^Ga-FAPI-04 PET is a reliable modality for monitoring the progression of BIPF. Therefore, molecular imaging of ^68^Ga-FAPI-04 has essential implications for noninvasive diagnosis of pulmonary fibrosis and evaluation of fibrotic activity.

Pirfenidone and nintedanib exert their anti-fibrotic effects through a dual mechanism involving the modulation of inflammatory cell activity and fibroblast function, coupled with the suppression of key inflammatory cytokines and profibrotic growth factors (35, 36). Our research indicates that ^68^Ga-FAPI PET can accurately reflect the changes in nintedanib treatment before and after pulmonary fibrosis, with consistent findings from both pathological and PET results. Therefore, ^68^Ga-FAPI-04 PET can play a significant role in monitoring treatment response.

Regarding Longitudinal evaluation of *ex vivo* and *in vivo* ^68^Ga- FAPI-04 uptake in murine pulmonary fibrosis models demonstrated a characteristic temporal uptake pattern. Additionally, SUVR may also have a significant impact on the results. In PET/CT imaging, SUVR is also one of the key parameters for probe evaluation (37). Additionally, we further explored the metabolic parameter SUVR of ^68^Ga-FAPI PET. Among the six datasets (Lung/Blood, Lung/Liver, Lung/Bone, Lung/Brain, Lung/Spleen, Lung/Muscle), significant differences in Lung/Blood ratios were observed across groups, with higher SUVR values appearing in the first week. For Lung/Liver ratios, the SUVR in the first week was the highest among all groups and showed statistically significant differences compared to the control group. Similarly, in the analysis of Lung/Muscle ratios, the uptake in the second week demonstrated statistically significant differences compared to the control group and Groups 1, 3, 4, and 5, with the highest uptake observed during this period. Based on these findings, we infer that the SUVR values for Lung/Blood, Lung/Liver, and Lung/Muscle peaked at the 1st, 1st, and 2nd weeks, respectively. Therefore, SUVR can also serve as a reliable parameter for evaluating fibrotic uptake patterns.

This study also has several limitations. First, our analysis of human data was limited to a brief retrospective summary. Incorporating prospective human studies in future research could enhance the clinical relevance of these findings. Second, compared to existing literature, the correlation analysis in this study requires further refinement. Third, the results of Masson’s immunohistochemistry were suboptimal. Fourth, clinically, BIPF during chemotherapy typically occurs in the context of malignancies such as lymphoma. However, our models were based on in BIPF normal mice, leading to inherent differences between our experimental setup and real-world clinical scenarios. Therefore, in future research, we need to address these aspects to improve our work further.

## 4 Conclusion

These findings underscore their translational relevance in clinical practice, particularly for early detection and prophylactic management of BIPF in lymphoma patients undergoing chemotherapy. Overall, our study demonstrated that ^68^Ga-FAPI-04 PET could be a potential tool to noninvasively diagnose BIPF, characterize the progression of pulmonary fibrosis, and assess the disease activity in murine models and humans.

## 5 Materials and methods

### 5.1 Animal experiments

**Figure 6:**
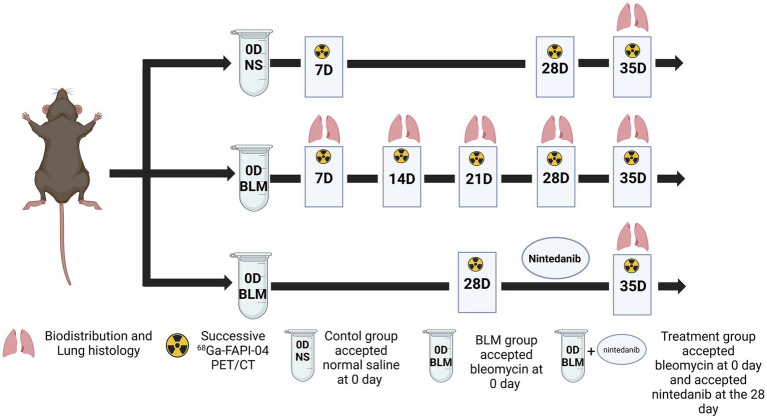
Animal grouping and experimental design. NS denotes the control group; BLM denotes mice receiving bleomycin for BIPF model establishment; another group of mice with BIPF was treated with nintedanib for treatment (Created by biorender).

Animal experiments were performed in accordance with the Guide and Use of Laboratory Animals and approved by the Institutional Animal Care and Use Committee of the Chongqing University Cancer Hospital. Six-week-old male C57/BL6 mice were randomly allocated into one of three experimental groups: control, bleomycin (BLM), and BLM + nintedanib. On the day (D) 0, the mice in the BLM and BLM + nintedanib. Groups received a single intratracheal injection of BLM (5 mg/kg) to establish the pulmonary fibrosis model, while the control group received saline. Mice in the BLM + nintedanib. Group received oral nintedanib (50 mg/kg/day) from day 28 after bleomycin instillation until the end of the experiment on day 35. Figure 6 shows the experimental schedule.

### 5.2 Radiolabeling of ^68^Ga-FAPI-04

The precursor FAPI-04 was purchased from MCE (MedChemExpress, USA), with a purity grade of 98% and a mass of 872.91. FAPI-04 radiolabeling was performed according to the following protocol: 50 μg of FAPI-04 was dissolved in 1 mL of sodium acetate solution (0.25 M), and 4 mL ^68^Ga-solution (approximately 1.7 GBq) was added to a pH of 3.3–3.6. The reaction mixture was heated for 10 min at 80°C, and the product was purified using a Sep-pak ^18^C column. It was then eluted with 1 mL 50% ethanol and 4 mL saline. Quality control was performed by radio-HPLC on an ^18^C reverse-phase column with a gradient elution of either H_2_O with 0.1% TFA (solvent A) or CH_3_CN with 0.1% TFA (solvent B). The mobile phase conditions were 0–50 min, 10–90% B, and 1 mL/min. The radiochemical purity of the final product was more than 95% and pyrogen-free.

### 5.3 PET Imaging and biodistribution studies

Micro-PET/CT (nanoScan PET/CT 82 s; Mediso Medical Imaging System Ltd., Hungary) imaging studies were conducted on control, bleomycin (BLM), and BLM + nintedanib mice. The mice were placed in the imaging chamber, anesthetized with a 2.0% isoflurane/oxygen gas mixture, and maintained under 1.5% isoflurane at a body temperature of 37°C throughout the imaging experiment. Static PET imaging was obtained about 50 min after intravenous injection of 0.18 ± 0.02 MBq/ g of ^68^Ga-FAPI. We performed 10-min static scans with the mice on a PET/CT equipped with computer-controlled vertical and horizontal chamber motion with an effective field of view (FOV) of 9.8 cm. After model initiation at day 0 (D0), the mice underwent longitudinal ^68^Ga-FAPI PET/CT at D7, D14, D21, D28 and D35. The SUVs of the tissues of interest (blood, brain, muscle, left lung, right lung, liver, spleen, kidney, and bone) were quantified from the micro-PET/CT data. Quantification of the images was performed via Carimas 2.10 software (Turke PET Centre, Turku, Finland). In the BLM group, 4 mice were sacrificed after 1 h injection at D7, D14, D21, D28 and D35. In the control and treatment group, 4 mice were sacrificed at D35. Major organs (blood, brain, heart, muscle, left lung, right lung, liver, spleen, kidney, muscle, and bone) were weighed and measured for radioactivity on a gamma counter using Automatic Gamma Counter (WIZARD^2^-2480, PerkinElmer Instruments Inc. USA). The concentration of radioactivity was determined as a percentage injected dose per gram (%ID/g), and the data were expressed as mean ± SD.

### 5.4 Hematoxylin–eosin (HE) staining, immunohistochemistry, and quantification

The lungs were fixed in 4% paraformaldehyde, dehydrated using gradient alcohol, embedded in paraffin, and cut into 5-μm slices for HE and FAP immunohistochemistry (IHC) staining. Staining results were observed under a slide scanner Pannoramic 250FLAS. Aipathwell software was used to quantify the percentage of positively stained areas. Detailed steps are provided in Supplementary materials.

### 5.5 Statistical analysis

Statistical analyses were performed using Prism 8.0 software (GraphPad Software, San Diego, CA, USA). Comparison between multiple groups was performed using the one-way ANOVA with Tukey’s *post hoc* test and Kruskal-Wallis test. A *p* < 0.05 was considered significant. Results are presented as median ± Standard Deviation.

## Data Availability

The datasets presented in this study can be found in online repositories. The names of the repository/repositories and accession number(s) can be found at: https://data.mendeley.com/preview/9hmypp2txx?a=b8b38f0d-9ac1-41cd-ac8e-53c6af3e0962.
