# Supplementary material for: 68Ga-FAPI PET/CT for diagnostic accuracy and therapeutic response assessment in bleomycin-induced pulmonary fibrosis: an integrated preclinical study
Source: Front Med (Lausanne). 2025 Jun 26;12:1613010. doi: 10.3389/fmed.2025.1613010 (PMC12240757; doi:10.3389/fmed.2025.1613010)
Supplement: Supplementary file 1 [file Table_1.DOCX]

**Table S1 The ^68^Ga-FAPI-04 SUVmean of left lung**

| Group | Mean ± SD | *P* value | | | | | |
| --- | --- | --- | --- | --- | --- | --- | --- |
|  |  | Vs. Contol | Vs. 1w | Vs. 2w | Vs. 3w | Vs. 4w | Vs. 5w |
| Contol | 0.11 ±0.02 | - | - | - | - | - | - |
| 1w | 0.16 ±0.07 | 0.9742 | - | - | - | - | - |
| 2w | 0.19 ±0.05 | 0.8146 | 0.9938 | - | - | - | - |
| 3w | 0.33 ±0.05 | 0.0426^*^ | 0.1162 | 0.2824 | - | - | - |
| 4w | 0.67 ±0.14 | ＜0.0001^****^ | ＜0.0001^****^ | ＜0.0001^****^ | 0.0004^***^ | - | - |
| 5w | 0.48 ±0.11 | 0.0004^***^ | 0.0009^***^ | 0.0026^**^ | 0.1979 | 0.0597 | - |

(**P* < 0.05, ***P* < 0.01, ****P* < 0.001, *****P*＜0.0001, Ordinary one-way ANOVA with Tukey's post hoc test )

**Table S2 The ^68^Ga-FAPI-04 SUVmean of right lung**

| Group | Mean ± SD | *P* value | | | | | |
| --- | --- | --- | --- | --- | --- | --- | --- |
|  |  | Vs. Contol | Vs. 1w | Vs. 2w | Vs. 3w | Vs. 4w | Vs. 5w |
| Contol | 0.12 ±0.03 | - | - | - | - | - | - |
| 1w | 0.12 ±0.04 | ＞0.9999 | - | - | - | - | - |
| 2w | 0.18 ±0.05 | 0.9650 | 0.9684 | - | - | - | - |
| 3w | 0.35 ±0.07 | 0.0553 | 0.0397^*^ | 0.1661 | - | - | - |
| 4w | 0.65 ±0.18 | ＜0.0001^****^ | ＜0.0001^****^ | ＜0.0001^****^ | 0.0032^**^ | - | - |
| 5w | 0.45 ±0.09 | 0.0028^**^ | 0.0016^**^ | 0.0077^**^ | 0.6172 | 0.0783 | - |

(**P* < 0.05, ***P* < 0.01, ****P* < 0.001, *****P*＜0.0001, Ordinary one-way ANOVA with Tukey's post hoc test )

**Table S3 Biodistribution in Murine Models of Bleomycin-Induced Pulmonary Fibrosis**

| Group  Tissues | 1w | 2w | 3w | 4w | 5w |
| --- | --- | --- | --- | --- | --- |
| Blood  Brain  Heart  Liver  Spleen  Lung(L)  Lung(R)  Kidney  Pancreas  Stomach  Colon  Muscle  Bone | 1.72 ± 1.56  0.19 ± 0.16  0.88 ± 0.71  0.71 ± 0.50  0.77 ± 0.54  1.89 ± 1.44  1.66 ± 1.22  11.55 ± 13.38  0.77 ± 0.75  0.71 ± 0.68  0.80 ± 0.73  0.93 ± 0.69  1.79 ± 2.36 | 2.17 ± 0.37  0.22 ± 0.15  1.64 ± 0.81  1.51 ± 0.76  1.68 ± 1.08  3.04 ± 2.26  2.14 ± 0.66  18.30 ± 17.02  0.95 ± 0.19  1.09 ± 0.36  0.99 ± 0.17  1.10 ± 0.48  2.63 ± 2.90 | 4.47 ± 3.18  0.28 ± 0.16  2.30 ± 1.15  1.69 ± 1.02  1.99 ± 0.94  3.37 ± 2.38  2.98 ± 2.19  41.97 ±46.41  1.92 ± 1.02  2.27 ± 1.56  1.99 ± 1.34  2.13 ± 0.86  5.74 ± 2.88 | 23.07 ± 13.51  1.04 ± 0.40  12.22 ± 6.70  7.09 ± 3.20  7.87 ± 4.22  17.49 ± 4.42  16.83 ± 5.69  237.95±60.20  10.04 ± 6.01  10.55 ± 6.15  9.31 ± 5.04  10.17 ± 5.09  13.01 ± 8.14 | 9.76 ± 5.98  0.43 ± 0.22  3.93 ± 1.96  3.53 ± 2.12  2.98 ± 1.25  6.18 ± 2.54  5.09 ± 2.09  37.40 ± 23.59  3.39 ± 1.83  3.97 ± 2.18  3.98 ± 1.51  2.72 ± 1.40  4.59 ± 1.72 |

**Table S4** **The Biodistribution of Left Lung**

| Group | Mean ± SD | *P* value | | | | | |
| --- | --- | --- | --- | --- | --- | --- | --- |
|  |  | Vs. Contol | Vs. 1w | Vs. 2w | Vs. 3w | Vs. 4w | Vs. 5w |
| Contol | 1.07 ±0.19 | - | - | - | - | - | - |
| 1w | 1.89 ±1.89 | 0.9998 | - | - | - | - | - |
| 2w | 3.04 ±2.26 | 0.9426 | 0.9863 | - | - | - | - |
| 3w | 3.37 ±2.38 | 0.8947 | 0.9582 | ＞0.9999 | - | - | - |
| 4w | 17.40 ±4.43 | ＜0.0001^****^ | ＜0.0001^****^ | ＜0.0001^****^ | ＜0.0001^****^ | - | - |
| 5w | 6.18 ±2.54 | 0.2458 | 0.2226 | 0.5242 | 0.6351 | 0.0004^***^ | - |

(*****P* < 0.0001, ****P* < 0.001, Ordinary one-way ANOVA with Tukey's post hoc test )

**Table S5 The Biodistribution of Right Lung**

| Group | Mean ± SD | *P* value | | | | | |
| --- | --- | --- | --- | --- | --- | --- | --- |
|  |  | Vs. Contol | Vs. 1w | Vs. 2w | Vs. 3w | Vs. 4w | Vs. 5w |
| Contol | 0.96 ±0.42 | - | - | - | - | - | - |
| 1w | 1.66 ±1.22 | 0.9991 | - | - | - | - | - |
| 2w | 2.14 ±0.66 | 0.9923 | 0.9999 | - | - | - | - |
| 3w | 2.98 ±2.19 | 0.9028 | 0.9762 | ＞0.9978 | - | - | - |
| 4w | 16.83 ±5.69 | ＜0.0001^****^ | ＜0.0001^****^ | ＜0.0001^****^ | ＜0.0001^****^ | - | - |
| 5w | 5.09 ±2.10 | 0.4122 | 0.5339 | 0.7285 | 0.8864 | 0.0008^***^ | - |

(*****P* < 0.0001, ****P* < 0.001, Ordinary one-way ANOVA with Tukey's post hoc test)

**Table S6 FAP IHC H-score Findings of BLM-Induced Lung Fibrosis**

| Group | Mean ± SD | *P* value | | | | | |
| --- | --- | --- | --- | --- | --- | --- | --- |
|  |  | Vs. Contol | Vs. 1w | Vs. 2w | Vs. 3w | Vs. 4w | Vs. 5w |
| Contol | 0.48 ±0.33 | - | - | - | - | - | - |
| 1w | 4.21 ±0.75 | 0.6660 | - | - | - | - | - |
| 2w | 4.86 ±0.73 | 0.1195 | 0.7698 | - | - | - | - |
| 3w | 5.14 ±0.69 | 0.0466^*^ | 0.4500 | 0.9910 | - | - | - |
| 4w | 7.77 ±0.37 | ＜0.0001^**^^**^ | 0.0001^***^ | 0.0007^***^ | 0.0017^**^ | - | - |
| 5w | 4.83 ±0.57 | 0.1288 | 0.7940 | ＞0.9999 | 0.9872 | 0.0007^***^ | - |

(**P* < 0.05, ***P* < 0.01, ****P* < 0.001, *****P*＜0.0001, Ordinary one-way ANOVA with Tukey's post hoc test )

**Table S7 FAP IHC Positive Area Ratio Findings of BLM-Induced Lung Fibrosis**

| Group | Mean ± SD | *P* value | | | | | |
| --- | --- | --- | --- | --- | --- | --- | --- |
|  |  | Vs. Contol | Vs. 1w | Vs. 2w | Vs. 3w | Vs. 4w | Vs. 5w |
| Contol | 2.22 ±0.36 | - | - | - | - | - | - |
| 1w | 2.27 ±0.63 | ＞0.9999 | - | - | - | - | - |
| 2w | 2.28 ±0.43 | ＞0.9999 | ＞0.9999 | - | - | - | - |
| 3w | 2.56 ±0.45 | 0.9734 | 0.9853 | 0.9832 | - | - | - |
| 4w | 3.55 ±0.73 | 0.1005 | 0.1180 | 0.0886 | 0.3208 | - | - |
| 5w | 1.89 ±0.67 | 0.9683 | 0.9479 | 0.9213 | 0.6359 | 0.0177^*^ | - |

(**P* < 0.05, ***P* < 0.01, ****P* < 0.001, *****P*＜0.0001, Ordinary one-way ANOVA with Tukey's post hoc test )

**Table S8 FAP IHC Area Density Findings of BLM-Induced Lung Fibrosis**

| Group | Mean ± SD | *P* value | | | | | |
| --- | --- | --- | --- | --- | --- | --- | --- |
|  |  | Vs. Contol | Vs. 1w | Vs. 2w | Vs. 3w | Vs. 4w | Vs. 5w |
| Contol | 0.48 ±0.33 | - | - | - | - | - | - |
| 1w | 4.21 ±0.75 | ＞0.9999 | - | - | - | - | - |
| 2w | 4.86 ±0.73 | 0.8739 | 0.9419 | - | - | - | - |
| 3w | 5.14 ±0.69 | 0.6671 | 0.7734 | 0.9947 | - | - | - |
| 4w | 7.77 ±0.37 | 0.0047^**^ | 0.0066^**^ | 0.0184^*^ | 0.0661 | - | - |
| 5w | 4.83 ±0.57 | 0.9908 | 0.9986 | 0.9961 | 0.9323 | 0.0130^*^ | - |

(**P* < 0.05, ***P* < 0.01, Ordinary one-way ANOVA with Tukey's post hoc test )

**Table S9 Comparison of SUVmean Values Among Therapy Groups**

| Group | Mean ± SD | *P* value | | |
| --- | --- | --- | --- | --- |
|  |  | Vs.NS | Vs.BLM | Vs.BLM+nintedanib |
| NS | 0.11 ±0.02 | - | - | - |
| BLM | 0.45 ±0.10 | ＜0.0001^****^ | - | - |
| BLM+nintedanib | 0.60 ±0.11 | ＜0.0001^****^ | 0.0323^*^ | - |

(**P* < 0.05, *****P* < 0.0001, Ordinary one-way ANOVA with Tukey's post hoc test )

**Table S10 Comparison of SUVR(Lung/Blood) Values Among Therapy Groups**

| Group | Mean ± SD | *P* value | | |
| --- | --- | --- | --- | --- |
|  |  | Vs.NS | Vs.BLM | Vs.BLM+nintedanib |
| NS | 0.67 ±0.05 | - | - | - |
| BLM | 1.07 ±0.11 | ＜0.0001^****^ | - | - |
| BLM+nintedanib | 0.85 ±0.11 | 0.0144^*^ | 0.0024^**^ | - |

(**P* < 0.05, ***P* < 0.01, *****P* < 0.0001, Ordinary one-way ANOVA with Tukey's post hoc test )

**Table S11 Comparison of SUVR(Lung/Liver) Values Among Therapy Groups**

| Group | Mean ± SD | *P* value | | |
| --- | --- | --- | --- | --- |
|  |  | Vs.NS | Vs.BLM | Vs.BLM+nintedanib |
| NS | 0.75 ±0.07 | - | - | - |
| BLM | 1.48 ±0.28 | ＜0.0001^****^ | - | - |
| BLM+nintedanib | 1.05 ±0.10 | 0.0279^*^ | 0.0020^**^ | - |

(**P* < 0.05, ***P* < 0.01, *****P* < 0.0001, Ordinary one-way ANOVA with Tukey's post hoc test )

**Table S12 Comparison of SUVR(Lung/Bone) Values Among Therapy Groups**

| Group | Mean ± SD | *P* value | | |
| --- | --- | --- | --- | --- |
|  |  | Vs.NS | Vs.BLM | Vs.BLM+nintedanib |
| NS | 1.30 ±0.08 | - | - | - |
| BLM | 2.04 ±0.56 | 0.0039^**^ | - | - |
| BLM+nintedanib | 1.46 ±0.09 | 0.6827 | 0.0209^*^ | - |

(**P* < 0.05, ***P* < 0.01, Kruskal-Wallis test )

**Table S13 Comparison of SUVR(Lung/Brain) Values Among Therapy Groups**

| Group | Mean ± SD | *P* value | | |
| --- | --- | --- | --- | --- |
|  |  | Vs.NS | Vs.BLM | Vs.BLM+nintedanib |
| NS | 1.80 ±0.47 | - | - | - |
| BLM | 4.19 ±1.41 | 0.0024^**^ | - | - |
| BLM+nintedanib | 2.97 ±0.31 | 0.0602 | 0.9127 | - |

(***P* < 0.01, *****P* < 0.0001, Kruskal-Wallis test )

**Table S14 Comparison of SUVR(Lung/Spleen) Values Among Therapy Groups**

| Group | Mean ± SD | *P* value | | |
| --- | --- | --- | --- | --- |
|  |  | Vs.NS | Vs.BLM | Vs.BLM+nintedanib |
| NS | 0.86 ±0.14 | - | - | - |
| BLM | 1.89 ±0.75 | 0.0037^**^ | - | - |
| BLM+nintedanib | 0.98 ±0.06 | 0.8828 | 0.0096^**^ | - |

(***P* < 0.01, Ordinary one-way ANOVA with Tukey's post hoc test )

**Table S15 Comparison of SUVR(Lung/Muscle) Values Among Therapy Groups**

| Group | Mean ± SD | *P* value | | |
| --- | --- | --- | --- | --- |
|  |  | Vs.NS | Vs.BLM | Vs.BLM+nintedanib |
| NS | 2.27 ±0.91 | - | - | - |
| BLM | 3.95 ±0.98 | 0.0167^*^ | - | - |
| BLM+nintedanib | 3.04 ±0.87 | 0.3452 | 0.2309 | - |

(**P* < 0.05, Ordinary one-way ANOVA with Tukey's post hoc test )

**Table S16 SUVR(Lung/Blood) Analysis in Bleomycin-Induced Pulmonary Fibrosis Mice Compared to Control Group**

| Group | Mean ± SD | *P* value | | | | | |
| --- | --- | --- | --- | --- | --- | --- | --- |
|  |  | Vs. Contol | Vs. 1w | Vs. 2w | Vs. 3w | Vs. 4w | Vs. 5w |
| Contol | 0.67 ±0.05 | - | - | - | - | - | - |
| 1w | 1.09 ±0.17 | ＜0.0001^****^ | - | - | - | - | - |
| 2w | 0.85 ±0.07 | 0.0358^*^ | 0.0018^**^ | - | - | - | - |
| 3w | 0.79 ±0.12 | 0.3884 | 0.0001^***^ | 0.8404 | - | - | - |
| 4w | 0.82 ±0.09 | 0.1487 | 0.0007^***^ | 0.9930 | 0.9909 | - | - |
| 5w | 0.84 ±0.13 | 0.0667 | 0.0021^**^ | ＞0.9999 | 0.9193 | 0.9987 | - |

(**P* < 0.05, ***P* < 0.01, ****P* < 0.001, *****P* < 0.0001, Ordinary one-way ANOVA with Tukey's post hoc test )

**Table S17 SUVR(Lung/Liver) Analysis in Bleomycin-Induced Pulmonary Fibrosis Mice Compared to Control Group**

| Group | Mean ± SD | *P* value | | | | | |
| --- | --- | --- | --- | --- | --- | --- | --- |
|  |  | Vs. Contol | Vs. 1w | Vs. 2w | Vs. 3w | Vs. 4w | Vs. 5w |
| Contol | 0.75 ±0.07 | - | - | - | - | - | - |
| 1w | 1.08 ±0.10 | ＜0.0001^****^ | - | - | - | - | - |
| 2w | 0.99 ±0.09 | 0.0006^***^ | 0.5256 | - | - | - | - |
| 3w | 0.99 ±0.10 | 0.0009^***^ | 0.4598 | ＞0.9999 | - | - | - |
| 4w | 1.04 ±0.07 | ＜0.0001^****^ | 0.9650 | 0.9175 | 0.8752 | - | - |
| 5w | 1.02 ±0.13 | ＜0.0001^****^ | 0.8721 | 0.9856 | 0.9707 | 0.9984 | - |

(**P* < 0.05, ***P* < 0.01, ****P* < 0.001, *****P* < 0.0001, Ordinary one-way ANOVA with Tukey's post hoc test )

**Table S18 SUVR(Lung/Bone) Analysis in Bleomycin-Induced Pulmonary Fibrosis Mice Compared to Control Group**

| Group | Mean ± SD | *P* value | | | | | |
| --- | --- | --- | --- | --- | --- | --- | --- |
|  |  | Vs. Contol | Vs. 1w | Vs. 2w | Vs. 3w | Vs. 4w | Vs. 5w |
| Contol | 1.30 ±0.08 | - | - | - | - | - | - |
| 1w | 0.88±0.13 | 0.0439^*^ | - | - | - | - | - |
| 2w | 1.28 ±0.40 | ＞0.9999 | 0.0460^*^ | - | - | - | - |
| 3w | 1.05 ±0.26 | 0.3823 | 0.8290 | 0.4152 | - | - | - |
| 4w | 1.61 ±0.12 | 0.1452 | ＜0.0001^****^ | 0.0863 | 0.0008^***^ | - | - |
| 5w | 1.18 ±0.16 | 0.9447 | 0.2519 | 0.9660 | 0.8889 | 0.0165^*^ | - |

(**P* < 0.05, ****P* < 0.001, *****P* < 0.0001, Ordinary one-way ANOVA with Tukey's post hoc test )

**Table S19 SUVR(Lung/Brain) Analysis in Bleomycin-Induced Pulmonary Fibrosis Mice Compared to Control Group**

| Group | Mean ± SD | *P* value | | | | | |
| --- | --- | --- | --- | --- | --- | --- | --- |
|  |  | Vs. Contol | Vs. 1w | Vs. 2w | Vs. 3w | Vs. 4w | Vs. 5w |
| Contol | 1.80 ±0.48 | - | - | - | - | - | - |
| 1w | 2.73±0.36 | 0.0064^**^ | - | - | - | - | - |
| 2w | 3.60 ±0.40 | ＜0.0001^****^ | 0.0065^**^ | - | - | - | - |
| 3w | 2.41 ±0.49 | 0.1076 | 0.7295 | ＜0.0001^****^ | - | - | - |
| 4w | 3.38 ±0.38 | ＜0.0001^****^ | 0.0698 | 0.9123 | 0.0006^***^ | - | - |
| 5w | 3.12 ±0.42 | ＜0.0001^****^ | 0.5214 | 0.2504 | 0.0197^*^ | 0.8260 | - |

(**P* < 0.05, ***P* < 0.01, ****P* < 0.001, *****P* < 0.0001, Ordinary one-way ANOVA with Tukey's post hoc test )

**Table S20 SUVR(Lung/Spleen) Analysis in Bleomycin-Induced Pulmonary Fibrosis Mice Compared to Control Group**

| Group | Mean ± SD | *P* value | | | | | |
| --- | --- | --- | --- | --- | --- | --- | --- |
|  |  | Vs. Contol | Vs. 1w | Vs. 2w | Vs. 3w | Vs. 4w | Vs. 5w |
| Contol | 0.86 ±0.14 | - | - | - | - | - | - |
| 1w | 0.91±0.06 | 0.9769 | - | - | - | - | - |
| 2w | 0.92 ±0.08 | 0.9414 | ＞0.9999 | - | - | - | - |
| 3w | 0.68 ±0.15 | 0.1001 | 0.0229^*^ | 0.0051^**^ | - | - | - |
| 4w | 1.18 ±0.07 | 0.0014^**^ | 0.0178^*^ | 0.0076^*^^*^ | ＜0.0001^****^ | - | - |
| 5w | 1.03±0.17 | 0.1348 | 0.5722 | 0.4899 | ＜0.0001^****^ | 0.3061 | - |

(**P* < 0.05, ***P* < 0.01, *****P* < 0.0001, Ordinary one-way ANOVA with Tukey's post hoc test )

**Table S21 SUVR(Lung/Muscle) Analysis in Bleomycin-Induced Pulmonary Fibrosis Mice Compared to Control Group**

| Group | Mean ± SD | *P* value | | | | | |
| --- | --- | --- | --- | --- | --- | --- | --- |
|  |  | Vs. Contol | Vs. 1w | Vs. 2w | Vs. 3w | Vs. 4w | Vs. 5w |
| Contol | 2.27 ±0.91 | - | - | - | - | - | - |
| 1w | 1.96±0.79 | 0.9720 | - | - | - | - | - |
| 2w | 3.94 ±1.19 | 0.0018^**^ | ＜0.0001^****^ | - | - | - | - |
| 3w | 2.00 ±0.49 | 0.9824 | ＞0.9999 | ＜0.0001^****^ | - | - | - |
| 4w | 2.23 ±0.15 | ＞0.9999 | 0.9785 | 0.0005^***^ | 0.9875 | - | - |
| 5w | 1.39±0.06 | 0.3073 | 0.7144 | ＜0.0001^****^ | 0.6268 | 0.2842 | - |

***P* < 0.01, ****P* < 0.001, *****P* < 0.0001, Ordinary one-way ANOVA with Tukey's post hoc test )
